# Neuroprotective Effects of Normobaric Hyperoxia and Transplantation of Encapsulated Choroid Plexus Epithelial Cells on The Focal Brain Ischemia 

**DOI:** 10.22074/cellj.2021.7204

**Published:** 2021-07-17

**Authors:** Maesumeh Eslami, Shahrbanoo Oryan, Mehdi Rahnema, Mohammad Reza Bigdeli

**Affiliations:** 1 Department of Animal Physiology, Faculty of Biological Sciences, Kharazmi University, Tehran, Iran; 2 Biology Research Center, Zanjan Branch, Islamic Azad University, Zanjan, Iran; 3 Department of Animal Sciences and Biotechnology, Faculty of Life Sciences and Biotechnology, Shahid Beheshti University, Tehran, Iran; 4 Inistitute for Cognitive and Brain Science, Shahid Beheshti University, Tehran, Iran

**Keywords:** Brain Ischemia, Choroid Plexus, Hyperoxia, Oxidative Stress

## Abstract

**Objective:**

Choroid plexus epithelial cells (CPECs) have the epithelial characteristic, produce cerebrospinal fluid, contribute to the detoxification process in the central nervous system (CNS), and are responsible for the synthesis and release of many nerve growth factors. On the other hand, studies suggest that normobaric hyperoxia (HO) by induction of ischemic tolerance (IT) can protect against brain damage and neurological diseases. We examined the effect of combination therapy of encapsulated CPECs and HO to protect against ischemic brain injury.

**Materials and Methods:**

In this experimental study, six groups of adult male Wistar rats were randomly organized: sham, room air (RA)+middle cerebral artery occlusion (MCAO), HO+MCAO, RA+MCAO+encapsulated CPECs, HO+MCAO+encapsulated CPECs, RA+MCAO+empty capsules. RA/HO were pretreatment. The CPECs were isolated from the brain of neonatal Wistar rats, cultured, and encapsulated. Then microencapsulated CPECs were transplanted in the neck of the animal immediately after the onset of reperfusion in adult rats that had been exposed to 60 minutes MCAO. After 23 hours of reperfusion, the neurologic deficit score (NDS) was assessed. Next, rats were killed, and brains were isolated for measuring brain infarction volume, blood-brain barrier (BBB) permeability, edema, the activity of superoxide dismutase (SOD), and catalase (CAT) and also, the level of malondialdehyde (MDA).

**Results:**

Our results showed that NDS decreased equally in HO+MCAO, RA+MCAO+encapsulated CPECs, and HO+MCAO+encapsulated CPECs groups. Brain infarction volume decreased up 79%, BBB stability increased, edema decreased, SOD and CAT activities increased, and MDA decreased in the combination group of HO and transplantation of encapsulated CPECs in the ischemic brain as compared with when HO or transplantation of encapsulated CPECs was applied alone.

**Conclusion:**

The combination of HO and transplantation of encapsulated CPECs for stroke in rats was more effective than the other treatments, and it can be taken into account as a promising treatment for ischemic stroke.

## Introduction

Cerebral ischemia is characterized by an occlusion of blood vessels that leads to interruption of positional blood flow to the brain and the lack of oxygen and glucose (1). Furthermore, in the stroke, depolarization of the neuronal membrane, the release of the neurotransmitter glutamate, and activation of receptor n-methyl-d-aspartate (NMDA), overloading calcium and the apoptosis occur (2). These incidences are related to enhanced reactive oxygen species (ROS) production that disturbs the antioxidant systems and results in an increase of inflammation and brain injury. Moreover, the blood-brain barrier (BBB) loses its integrity due to reperfusion, the sudden increase in oxygen, extra production of ROS, and destruction of proteins of the blood vessel cell membrane (3-5). Therefore, by increasing the antioxidant capacity, it is expected to decrease brain tissue damage due to oxidative stress in stroke and improve the permeability of the BBB.

The exposure to under-threshold injurious stimuli induces ischemic tolerance (IT), also known as ischemic preconditioning (IP), that actives endogenous neuronal protective processes (6). Various stressors, including anesthetics, cortical spreading depression, ischemia, seizures, inflammatory mediators, and metabolic occlusive, can induce preconditioning in the brain (7). The evidence suggests that ROS mediate brain damage in cerebral mortal and sub-mortal ischemia importantly. Several studies propose that preconditioning with hyperoxia (HO) decreases ischemic brain injury mediated by induction of IT and via the production of ROS (8, 9) and is neuroprotective in experimental ischemic stroke (8-12). Moreover, reports show that HO is applied in the treatment of human stroke, also (13, 14).

**Fig.1:**
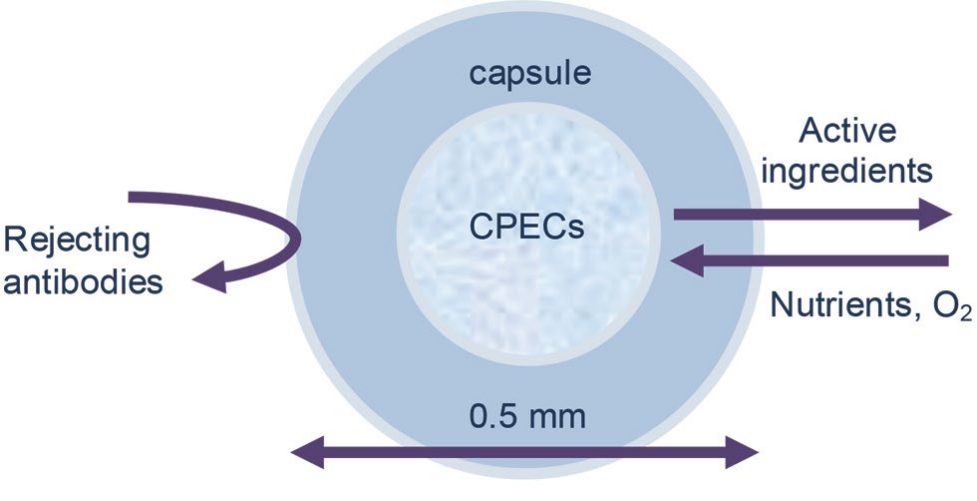
Schematic image of the encapsulated CPECs. The capsule prevents the immune system activation against CPECs and provides the interaction of cells with the extracellular environment. The diameter of the microcapsule is 0.5 mm. CPECs; Choroid plexus epithelial cells.

There are also many reports of cell therapy for stroke, which demonstrate cell transplantation has good functional and structural results in animals and humans (15-17). The choroid plexus (CP) is within the brain ventricles and consists of epithelial cells that are involved in the secretion of cerebrospinal fluid (CSF) and surround a weak connective tissue containing penetrable capillaries and cells of lymphoid family. Abundant neurotrophic factors, including nerve growth factor (NGF), brain-derived neurotrophic factor (BDNF), vascular endothelial growth factor (VEGF) neurotrophin 3-4 (NT3-4) and fibroblast growth factor 2 (FGF2) are produced and secreted by the choroid plexus epithelial cells (CPECs) to CSF (18, 19). On the other hand, it has been reported that encapsulation of cells by biomaterials such as alginate allows oxygen and nutrients to nourish the encapsulated cells and provides controlled diffusion of proteins and other therapeutic molecules and at the same time restricts the passage of cytotoxic agents from the host immune defense system (Fig.1) (20). *In vitro* studies show that many active neurotrophic factors such as BDNF and GDNF are secreted by both non-encapsulated and encapsulated CPECs that have a similar model of secretion (21). Therefore, it is expected that CPECs act indirectly by secreting and releasing the trophic substances without the encapsulation effect on the secretory property of cells. The neuroprotective effect of CPECs and conditioned medium of cultured CPECs against ROS-induced oxidative stress has been well shown (22). The previous studies indicated that transplantation of CPECs protected against ischemic brain injury and improved behavioral deficits in animal models of stroke (16, 23).

Although the effect of different monotherapies on stroke has been widely investigated, functional recovery is typically only partial. It seems that applying monotherapies in combination with each other to be an appropriate strategy to achieve a favorable recovery. Hence, in this context, we combined two therapeutic strategies to promote functional recovery after middle cerebral artery occlusion (MCAO) and investigated the effect of this combination on focal brain ischemia. Thus, considering the above reported beneficial effects of both HO and CPECs in the treatment of stroke, and considering that the combination of these two for the treatment of stroke had not been studied, we combined preconditioning by HO and transplants of encapsulated rat CPECs for ischemic stroke in rat for obtaining protective effects.

## Materials and Methods

### Experimental animals

In this experimental study, adult male Wister rats weighted from 250 up to 350 g were kept at fixed humidity, the temperature of 23 ± 2˚C and 12-hour cycle of light-dark (07:00-19:00) for all experiments. Water and food were accessible free. The conduction of all manners was with the approval of the Institutional Animal Ethics Committee of Islamic Azad University (code: IR.IAU.Z.REC.1396, 69). In total, five main groups (in each group, 35 rats) and a sham group (n=21 rats) of rats were randomly formed. HO+MCAO group was put up in an environmental chamber and subjected to 95% oxygen (O_2_) periodically, i.e., four sequential hours in each day for continuous six days (8). RA+MCAO group was located in the environmental chamber in the like procedure and subjected to room air (RA) equivalent (21% O_2 _) for the same intervals. The balance gas when using 95% O_2_ and 21% O_2_ , was nitrogen. Soda-lime, a CO_2_ absorber, was used at the bottom of the container to prevent CO_2_ retention. So with this, we tried to have the rest of nitrogen. The RA+MCAO+encapsulated CPECs group was exposed to RA, and alginate encapsulated CPECs were transplanted in the neck of the rats. The HO+MCAO+encapsulated CPECs group was subjected to HO for the same intervals, and then alginate encapsulated CPECs were implanted in the neck of the rats. The RA+MCAO+empty capsules group was exposed to RA, and free-cell capsules were transplanted in the neck of the rats. At 48 hours after pretreatment by the HO or RA, all the groups were exposed to MCAO for 60 minutes, and then encapsulated CPECs or empty capsules were transplanted in related groups. Each main group was divided randomly into four subgroups to evaluate infarct volume (n=7), brain water content or edema (n=7), the permeability of BBB (n=7), and catalase (CAT) and superoxide dismutase (SOD) activities and malondialdehyde (MDA) level (n=7). In the sham group, three subgroups were designed to evaluate cerebral edema (n=7), the permeability of BBB (n=7), and antioxidant activity (n=7). The neurobehavioral studies were performed by the individual blinded to animal groups before each group was divided randomly into the mentioned subgroups. In a subset of rats, just before removing animals from the environmental chamber, an analysis of arterial blood gas was carried out. Laser Doppler flowmeter (MBF3, Moor Instrumenats, Axminster, UK) was applied to the record of regional cerebral blood flow (rCBF).

### Choroid plexus epithelial cells isolation and culture

CP tissues were excised from the lateral ventricles of neonatal rats (4-5 day-old), rinsed by phosphate-buffered saline (PBS, Sigma, USA), and next, the incubation with 0.25% trypsin solution (Invitrogen-Gibco, England) was performed for 20 minutes at 37˚C. The next step was the addition of fetal bovine serum (FBS, Invitrogen-Gibco, England) and the centrifuge for 5 minutes. The sediment was transmitted to a culture medium, including Dulbeccoʼs Modified Eagleʼs Media (DMEM/F12, Invitrogen-gibco, England), 10% FBS, and 1% pen/strep antibiotic (Sigma, USA). 20 μM cytosine arabinoside (Sigma, USA) was used to prevent fibroblast proliferation for one week. The culture medium was changed every 48 hours (22).

### Immunocytofluorescence

Transthyretin (TTR) is the first known protein synthesized solely by the CP and is a marker for CPECs. In this study, for confirmation that cells isolated from the brain are CPECs and not another cell, the immunocytofluorescence was performed to identify the TTR marker. Immunocytofluorescence technique was performed according to the method previously described (22). A 24-well plate was used to CPECs culture. Then, cells were fixed with 4% paraformaldehyde (Sigma, USA). The next steps were the washing with PBS, to be permeable with Triton Χ-100 (Sigma, USA), and the incubation with the normal goat serum (Abcam, England). Afterward, cells were incubated with the primary antibody against TTR at 4˚C. After washing with PBS, the fluorescent secondary antibody (Abcam, England) was used. Finally, the cells were painted by diamidino-2- phenylindole (DAPI) dye (Sigma, USA) and studied by fluorescence microscopy (Olympus, IX 71, Japan).

### Preparing alginate encapsulated choroid plexus epithelial cells

The suspension of CPECs, separated from the bottom of the flask by trypsin, was prepared in a 1.5% alginate solution (Sigma, USA). Then, to the formation of alginate beads, suspension exuded to 60 mM CaCl_2_ solution (Merck, Germany). After that, incubation of alginate beads was performed respectively with 0.1% poly-L-lysine solution (Sigma, USA), 0.1% alginate solution, and 55 mM sodium citrate solution (Merck, Germany) each for 5 minutes (24). After washing microcapsules by the normal saline, medium of DMEM/F12 and FBS was used for the culture of microcapsules for seven days prior to implantation. Microcapsules were 0.5 mm in diameter (Fig.1).

### Studying the permeability of alginate microcapsules

For confirmation that the secreted material from the cells and the nutrients for the cells can pass through the microcapsule wall, fluorescent Thioflavin T (ThT, Sigma, USA) color was used. It can pass through the microcapsule wall and observe inside it. For this purpose, cell-free alginate microcapsules were created. Then, alginate microcapsules were incubated for 8 hours in a 0.4 mM ThT solution in the dark. Eventually, the washed microcapsules were studied by a fluorescent microscope (Olympus, IX 71, Japan).

### Environmental chamber

HO treatment (95% O_2_) was initiated in a chamber (65×35×45 cm) with a port of gas entry and exit. Oxygen was delivered at a rate of 3 L/minutes, constantly monitoring its concentration inside the container via an oxygen meter (Lutron-Do5510, Taiwan). A carbon dioxide absorber, Soda-lime (BDH Limited, Poole, UK), was used in the under of the container. According to the experimental groups, the oxygen concentration was kept at 95% or 21% for HO or RA groups, respectively.

### Focal cerebral ischemia and middle cerebral artery occlusion

**Fig.2:**
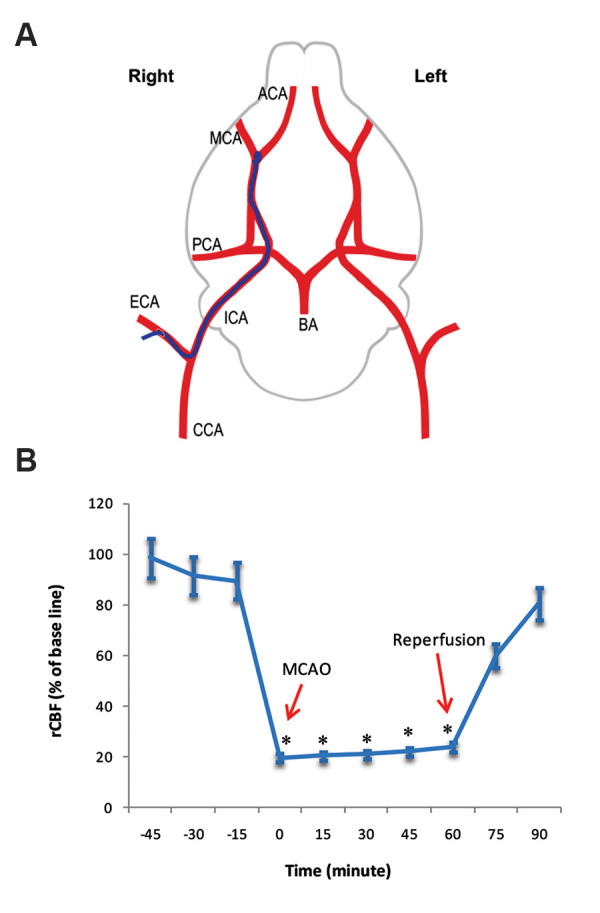
Middle cerebral artery occlusion and evaluation of the regional cerebral blood flow. **A.** Schematic presentation of MCAO in the right hemisphere of the rat brain. A nylon suture with the rounded head (blue filament) was inserted into the right ICA via the right ECA and blocked right MCA. **B.** The rCBF in rats undergoing MCAO surgery. The rCBF was characterized by 100% of a baseline before MCAO, but it decreased significantly during MCAO. The rCBF after reperfusion came close to the baseline. *; P<0.05 vs. before MCAO (n=7). MCAO; Middle cerebral artery occlusion, CCA; Common carotid artery, ICA; Internal carotid artery, ECA; external carotid artery, BA; Basilar artery, PCA; Posterior cerebral artery, MCA; Middle cerebral artery, ACA; Anterior cerebral artery, and rCBF; Regional cerebral blood flow.

For the anesthetization of rats, the 10% chloral hydrate (Merck, Germany, 350 mg/kg, i.p.) was used. According to the previously described method (25), MCAO was done. First, the right common carotid artery (CCA) was represented and separated. A 3-0 silicone-coated nylon suture with a rounded tip by heat was inserted into the internal carotid artery (ICA) and then was moved forward until it occluded the beginning of the middle cerebral artery (MCA). After the advance of approximately 20-22 mm of the suture, calculated from the carotid bifurcation, was created moderate resistance that showed the inhabitancy of the tip in the anterior cerebral artery (ACA) and the blocking the blood flow to the MCA (Fig.2A). After 60 minutes of ischemia, the suture was pulled out, and reperfusion was created. During surgery, rectal temperature was recorded with a thermometer (Citizen-513w, CITIZEN, UAE) and kept at 37˚C by heating and cooling of the surface.

### Transplantation of alginate microcapsules

At one hour after ischemia and immediately after the onset of reperfusion, the encapsulated CPECs or empty capsules were transplanted in the neck of the animal where the carotid artery was exposed to view. Afterward, the surgical site was sutured.

### Biocompatibility of the microcapsule

After 24 hours, microcapsules containing CPECs were regained to study the biocompatibility and stability of them when transplanted in the neck of the animal. Microcapsules were examined after washing with saline buffer under the inverted microscope. Also, to determine the percentage of live cells, decapsulation was initially performed with 55 mM sodium citrate solution. Then, 20 μl of cell suspension with 20 μl of 0.25% trypan blue dye was mixed. Afterward, 10 μl of the above mixture was placed on one side of a hemacytometer counter, and then cells studied by a light microscope. Blue cells are the dead cells, and clear cells are viable. The viable cell percentage was obtained via division of the number of viable cells to the number of total cells and multiplication by 100.

### Neurobehavioral evaluation

At 24 hours after pulling out the suture and while each rat was kept in a separate cage, the neurologic behaviors were assayed by an investigator blind to the experimental groups and endpoint assessment as follows (26): usual locomotion activity=score 0; bend of contralateral forelimb while the animal was suspended by the tail=score 1; contralateral rotational movement but usual state at relaxation=score 2; absence of righting reflex=score 3; and lack of automatic locomotion function=score 4. The rats that died less than 24 hours after surgery, their brains were colored. If the death was because of the subarachnoid hemorrhage or pulmonary insufficiency and asphyxia, they were omitted from the examination.

### Infarction volume evaluation

At 24 hours after reperfusion, animals were killed with chloral hydrate (Merck, Germany, 700 mg/kg, i.p.), and the decapitation was done. Then, rapid removing of brains and cooling in 4˚C normal saline for 15 minutes were performed. Brain, coronal sections were created with a thickness of 2 mm by using Brain Matrix (Tehran, Iran). The sections were soaked in a 2% solution of 2, 3, 5- triphenyl tetrazolium chloride (TTC, Merck, Germany) and immediately held at 37˚C for 15 minutes. Then, the photoinitiator of slices was carried out via a digital camera (Canon, DSC-W310). Finally, the infarct volume was calculated using UTHSCSA Image Tools image analysis software and pursuant to the manner of Swanson et al. (27) as follows: measuring of the colorless (infarct area) and colored areas in each hemisphere of the section, multiplying by the thickness 2 mm and then summation all of the sections: (corrected infarct volume)=(left hemisphere volume)−(right hemisphere volume−infarct volume).

### Assessment of brain water amount

First, the decapitation was performed. Then, brains were removed, and after separation of the cerebellum, pons, and olfactory bulb, wet weight (WW) was measured. Dry weight (DW) was assayed after 24 hours and subjected to 120˚C. The amount of brain water was obtained as [[(WW−DW)/WW]×100]

### Assessment of permeability of the blood-brain barrier

The stability of the BBB was investigated by studying Evans Blue (EB, Sigma Chemicals, USA) ejection (8). Briefly, a 2% EB solution (4 ml/kg) was injected in animal tail vein 30 minutes after MCAO. At 24 hours after reperfusion, anesthesia, and opening the thoracic cavity was done. Then, animals were transcardially perfused with 250 ml normal saline until the coming out of colorless perfusion fluid from the atrium. In this way, intravascular EB was washed out. Afterward, rats were decapitated, and the brain hemispheres were removed and weighed. 2.5 ml PBS was used for the homogenization of each hemisphere and extraction of EB. For the precipitation of the protein contents, 60% trichloroacetic acid (Merck, Germany, 2.5 ml) was added to the homogenized mixture and then mixed by vortex for 3 minutes. The next steps were included keeping the samples at 4˚C for 30 minutes, centrifugation at 1000×g for 30 minutes, and measuring the amount of EB in the supernatant at 610 nm wavelength using spectrophotometry (Genesys 5, USA). The amount of EB was shown as µg/g of brain tissue for a standard curve. 

### Extraction of protein from brain samples 

1ml buffer containing 0.32 mol/l sucrose, 1 mmol/l EDTA, and 10 nmol/l Tris-HCl, pH=7.4 was used for the homogenization of brain right hemisphere tissue (150 to 200 mg). The homogenized mixture was centrifuged at 13600×g for 30 minutes. Then, the supernatant was used for the measurement of SOD and CAT activities, MDA level, and protein contents (28). The measuring protein was done in agreeing to Bradford (29).

### Measuring the activity of superoxide dismutase, catalase and malondialdehyde level

The activity of SOD was determined according to the previous method (30) with some alteration. For obtaining a volume of 1 ml of the final assay mixture, 20 µl enzymatic extract was mixed with 50 mM sodium phosphate buffer (PB), pH=7.0, 0.1 mM EDTA, and 0.48 mM pyrogallol. The blank was a mixture of the above components, except enzymatic extract. The absorbance changes of the final assay mixture were recorded at 420 nm for 1 minute at 25˚C versus blank. The results were represented as U/mg protein. For measuring CAT activity, a volume of 1ml of the final assay mixture was prepared. For this purpose, 20 µl enzymatic extract was mixed with 50 mM PB, pH=7.0, and 10 mM hydrogen peroxide. The blank was a mixture of the above components, except enzymatic extract. Then, absorbance decrease was pursued at 240 nm wavelength for 1 minute at 25˚C versus blank. The amount of CAT activity was stated as U/mg protein. The level of MDA in homogenates was determined by applying the method described by Uchiyama and Mihara (31). 0.5 ml homogenate was mixed with 1% phosphoric acid solution (3 ml) and 0.6% thiobarbituric acid solution (1 ml). The next steps were included heating the mixture in a bain-marie at 95˚C for 45 minutes, cooling, adding n-butanol (4 ml), and mixing the solution by vortex, centrifugation at 3000×g for 10 minutes and measuring the absorbance of the supernatant at 532 nm. The standard was tetraethoxypropane (Merck, Germany). The concentrations of MDA were represented as nmol/mg protein.

### Measuring regional cerebral blood flow

Recording rCBF was performed by Velocitometry Laser Doppler flowmeter (MBF3D, Moor Instrument, Axminster, UK) (32). By placing the probe of laser Doppler flowmeter in the surface, Doppler flux was continually assessed from the 30 minutes before MCAO until 30 minutes after reperfusion.

### Statistical analysis

The data were presented as means ± SD and compared via one-way ANOVA followed by LSD. Mann-Whitney U test was used for the analysis of neurologic deficit score (NDS). The level of the statistical significance was set at P<0.05.

## Results

### Experimental conditions parameter

The pressure of CO_2_ and O_2_ analysis in the arterial blood showed that preclinical HO and RA were rightly created in the pretreatment groups. Any significant difference in pH and pressure of CO_2_ in HO and RA groups was not seen (P>0.05, pH=7.3 ± 0.09, 7.4 ± 0.1, pressure of CO_2_ : 39.2 ± 1.4, 42.8 ± 0.8), but the difference in the pressure of O_2_ in HO and RA groups was significant (P<0.001, 335 ± 24.7, 95.8 ± 6.9). rCBF was decreased to less than 24% of the baseline during MCAO in groups exposed to ischemia when compared with rCBF before ischemic damage (P<0.05, Fig.2B).

### Culture and identification of choroid plexus epithelial cells

**Fig.3:**
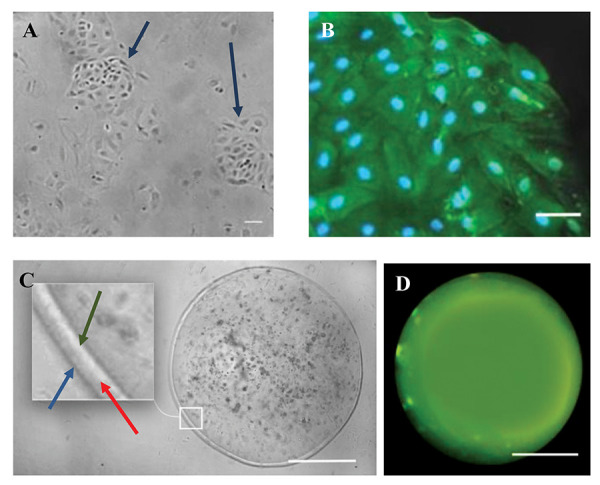
Recognition of choroid plexus epithelial cells (CPECs) and image of the microcapsule’s surface by fluorescence microscopy. **A.** The appearance of the CPECs 5 days after culturing. Arrows indicate clusters of polygonal cells (CPECs) in culture medium. **B.** The CPECs with maximum confluence (80%). The result of CPECs immunocytochemistry was positive for Transthyretin (TTR, green) (scale bar: 100 μm). **C. **Three layers of alginate microcapsule. Inner layer; Alginate (green arrow), Middle layer; Poly-L-Lysine (red arrow), and Outer layer; Alginate (blue arrow). **D. **Free-cell alginate microcapsule was incubated with ThT (scale bar: 200 μm).

Polygonal cells were observed in the flasks five days after CPECs culture, and their density was 15% (Fig.3A). Two weeks later, the total surface of the flasks was filled with cells that had an epithelial appearance. The immunocytofluorescence was performed to identify the TTR marker (Fig.3B).

### Alginate microcapsules

The results showed that the alginate microcapsules surface includes three layers of alginate, poly-L-lysin, and alginate (Fig.3C). When the empty alginate microcapsules were incubated with the ThT solution, ThT could permeate them via the pores on the surface of microcapsules (Fig.3D). Also, the surface of microcapsules containing CPECs did not change after 24 hours and was smooth as well as about 75- 80% of the cells in the microcapsule were alive. This suggests the biocompatibility and stability of the microcapsules and shows that CPECs indirectly and possibly by releasing trophic factors are effective and do not migrate themselves.

### Neurologic deficit scores

Median NDSs in the RA+MCAO group in comparison with sham was significantly different (2 vs. 0, P<0.05). Median NDSs in the HO+MCAO, RA+MCAO+encapsulated CPECs, and HO+MCAO+encapsulated CPECs groups decreased significantly in comparison with the RA+MCAO and RA+MCAO+empty capsules groups (1 vs. 2, P<0.05, Table 1).

### Infarction volume was decreased by hyperoxia and encapsulated choroid plexus epithelial cells

**Fig.4:**
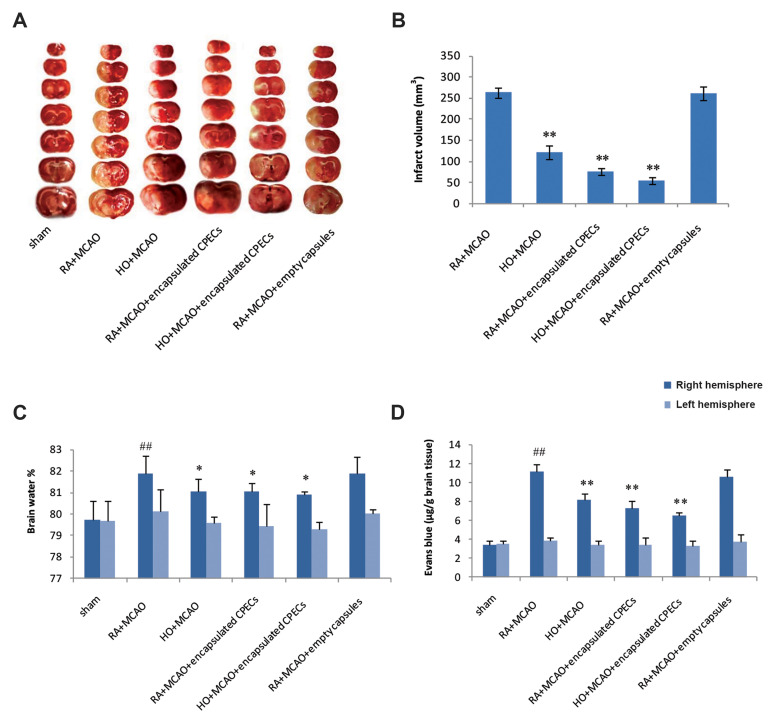
The effect of HO and CPECs on brain infarct volume, brain water content, and EB concentration in the experimental groups. **A. **The columns show rat brain coronal sections in the experimental groups. **B, C,** and **D.** ^##^; P<0.01 vs. sham, * ; P<0.05, **; P<0.01 vs. RA+MCAO group (n=7). RA; Room air, MCAO; Middle cerebral artery occlusion, HO; Hyperoxia, CPECs; Choroid plexus epithelial cells, and EB; Evans blue.

The infarct volume decreased in HO+MCAO, RA+MCAO+encapsulated CPECs, and HO+MCAO+encapsulated CPECs groups compared to the RA+MCAO group after 24 hours MCAO, significantly (P<0.01). Also, a significant difference was not observed between RA+MCAO and RA+MCAO+empty capsules groups in infarct volume (P>0.05, Fig.4A, B). 

### Hyperoxia and encapsulated encapsulated choroid plexus epithelial cells decreased brain edema and ameliorated blood-brain barrier permeability

The brain water amount increased in ischemic cerebral tissue (right hemisphere) in the RA+MCAO group compared to the right hemisphere of the sham group, significantly (P<0.01). The edema in the right hemisphere decreased significantly in HO+MCAO (P=0.043), RA+MCAO+encapsulated CPECs (P=0.039), and HO+MCAO+encapsulated CPECs (P=0.014) groups in comparison with a RA+MCAO group (P<0.05). Free-cell capsules had no effect (Fig.4C).

The results showed EB concentration in ischemic cerebral tissue (right hemisphere) in the RA+MCAO group increased and had a significant difference with the sham group (P<0.01). EB leakage in the right hemisphere reduced significantly in HO+MCAO, RA+MCAO+encapsulated CPECs, and HO+MCAO+encapsulated CPECs groups compared with the RA+MCAO group (P<0.01). Changes in BBB permeability in the left hemisphere were not significant. Also, Free-cell capsules had no effect (Fig.4D).

**Table 1:** Neurologic deficit scores (NDS) in the experimental groups


Number	Groups	NDS in each group					Premature death number	Total	Median	Statistical results
		0	1	2	3	4				

1	Sham	21	0	0	0	0	0	21	0	1 vs. 2= significant
2	RA+MCAO	0	4	13	3	8	6	28	2	3 vs. 2= significant
3	HO+MCAO	9	6	13	0	0	3	28	1	4 vs. 2= significant
4	RA+MCAO+encapsulated CPECs	12	8	8	0	0	2	28	1	5 vs. 2= significant
5	HO+MCAO+encapsulated CPECs	11	12	5	0	0	1	28	1	6 vs. 2= nonsignificant
6	RA+MCAO+empty capsules	0	1	14	4	9	5	28	2	


Results between groups analyzed with a significant level of P<0.05.

### Hyperoxia and encapsulated choroid plexus epithelial cells increased superoxide dismutase and catalase activity and decreased malondialdehyde level

**Fig.5:**
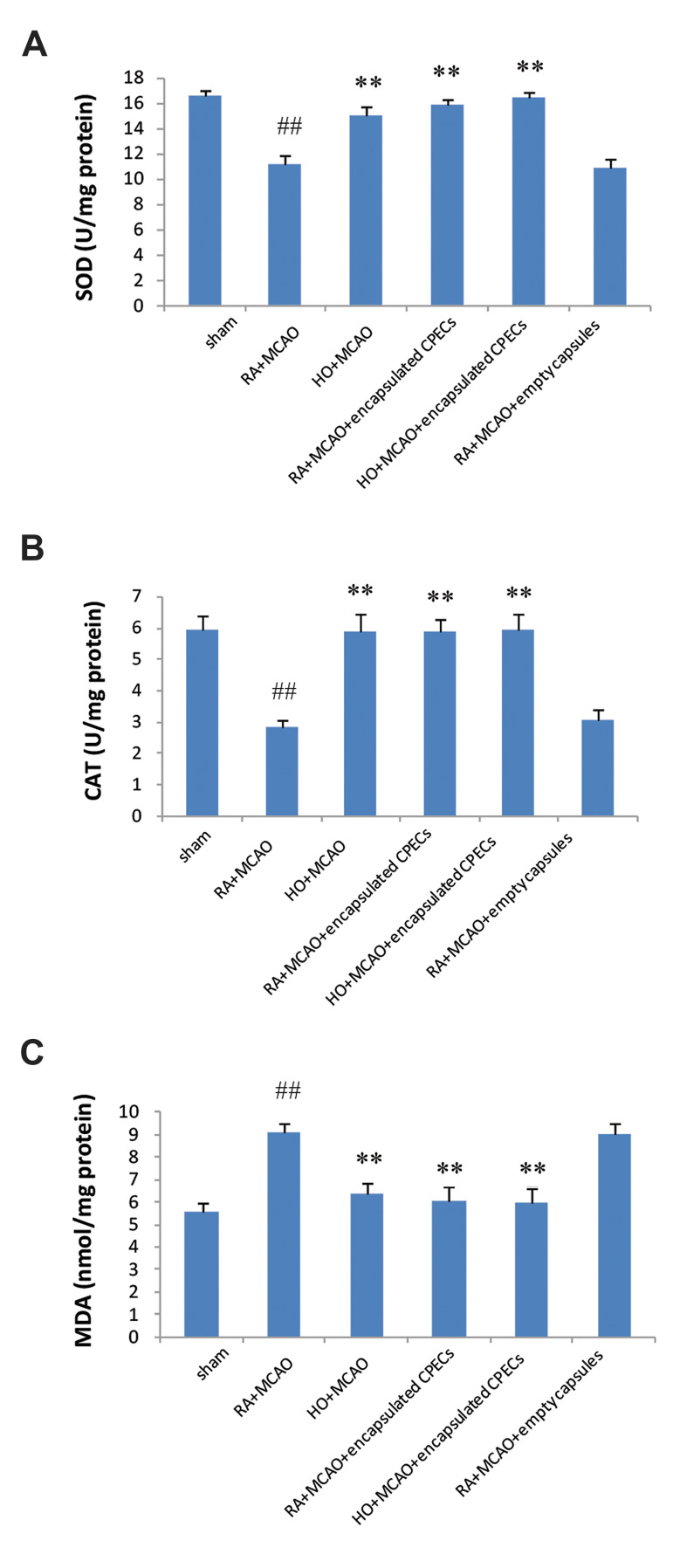
Evaluation of antioxidant capacity in the right hemisphere of experimental groups. **A. **The activation of SOD, **B.** CAT, and **C.** MDA level. ^##^; P<0.01 vs. sham group, **; P<0.01 vs. RA+MCAO group (n=7), SOD; Superoxide dismutase, CAT; Catalase, MDA; Malondialdehyde, RA; Room air, MCAO; Middle cerebral artery occlusion, HO; Hyperoxia, and CPECs; Choroid plexus epithelial cells.

At 24 hours after ischemia-reperfusion, SOD activity in the right hemisphere showed a decrease in RA+MCAO and RA+MCAO+empty capsules groups compared to the sham group (P<0.01). This value in the RA+MCAO group was 11.14 U/mg protein, whereas, in the HO+MCAO, RA+MCAO+encapsulated CPECs and HO+MCAO+encapsulated CPECs groups showed a significant increase, up to 15, 15.8 and 16.7 U/mg protein, respectively (P<0.01, Fig.5A). CAT activity in the right hemisphere decreased in the RA+MCAO and RA+MCAO+empty capsules groups in comparison to the sham group (P<0.01). But, it increased significantly in HO+MCAO, RA+MCAO+encapsulated CPECs, and HO+MCAO+encapsulated CPECs groups compared to the RA+MCAO group (P<0.01, Fig.5B).

Lipid peroxidation was indicated by the MDA content in the brain. MCAO increased the MDA level significantly in the right hemisphere in comparison to the sham group (P<0.01). However, the MDA amount in the right hemisphere lowered significantly in the HO+MCAO, RA+MCAO+encapsulated CPECs, and HO+MCAO+encapsulated CPECs groups compared to the RA+MCAO group (P<0.01, Fig.5C).

## Discussion

Brain ischemia is one of the outstanding causes of death throughout the world. Despite cerebral ischemia-associated high costs, there are limited treatment options against ischemic brain damage that most of them have failed to increase recovery rate following the induction of stroke, which proposes an urgent need for the development of new therapies for brain ischemia (1). 

Here, for the first time, we applied HO and transplantation of encapsulated CPECs of neonatal rats in combination with each other to enhance recovery and decrease symptoms in an MCAO model of cerebral ischemia in adult rats. In this work, results showed HO and encapsulated CPECs alone and, in combination with each other, had protective effects on oxidative stress-induced ischemia-reperfusion injury in rat brain and reduced brain damage. Also, results showed that HO and encapsulated CPECs alone and in combination with each other due to increasing antioxidant activity (increase SOD and CAT activities and decrease MDA) decreased edema, BBB permeability, neurologic deficits and brain damage in a rat model of MCAO.

Our data showed when HO was combined with encapsulated CPECs for cerebral ischemia, a significant reduction in infarction volume of 79% was achieved. Also, results demonstrated 53% and 70% reductions in total infarction volume by preconditioning with HO alone or transplantation of encapsulated CPECs alone, respectively. Combination therapy was effective than the HO and encapsulated CPECs alone. On the other hand, NDS, a marker of neurological behavior, was improved by HO, encapsulated CPECs, and an combination of HO and encapsulated CPECs, and all three treatments were equally effective as a neuroprotectant based on NDS. This equivalent effect may be explained as perhaps 24 hours is not enough for the effect of encapsulated CPECs on NDS and functional recovery and may increase with time to spare. In an experimental study, was shown normobaric HO decreased infarct volume and NDS in the MCAO model of stroke in rats that is in agreement with our results (33). Also, Borlongan et al. (23) showed improvement in behavioral functions and reduction of infarction volume by CPECs transplants three days after MCAO. 

According to experimental reports, transplants of encapsulated CPECs are more effective than the nonencapsulated CPECs transplants, and there is a major immune response to capsule-free CPECs in comparison with encapsulated CPECs. In addition, empty capsules have no effect on the improvement of stroke. Therefore, it may be said that the capsule has no protective effect alone, but it can enhance CPECs effects by reducing the reaction of the host. Our results showed that the viability of encapsulated CPECs after one day was 75-80%, and empty capsules had no therapeutic effect that is in agreement with the work of Borlongan et al. (23).

The interchange between blood and brain tissue is controlled by BBB. Disruption of BBB following the stroke increases edema and causes ischemic injury and mortality. In an experimental stroke, the protection of endothelial cells and the inhibition of MMP activity via SOD keeps the BBB integrity and decreases brain damage (34). We showed that EB concentration decreased, the stability of the BBB increased, and edema decreased in HO, encapsulated CPECs, and the combination groups when compared with RA+MCAO group, significantly. Moreover, the HO+MCAO+encapsulated CPECs group had more effect in reducing edema than the HO and encapsulated CPECs alone. The previous investigations indicated that reduction of brain edema and decrease of BBB permeability could occur by the HO in rats (8, 35). So far, there has been no report on the effect of the CPECs on edema and the BBB damage caused by stroke.

The evidence proposes that ROS-induced oxidative stress results in the injury of cellular macromolecules, which are linked to the death of neurons induced by ischemia-reperfusion injury (5). We showed that the activity of CAT and SOD increased in HO, encapsulated CPECs, and combination therapy groups when compared with the RA+MCAO group. The lipid peroxidation is determined via the MDA level. The concentration of MDA reverberates that the cause of brain damage is ROS. Extra ROS is scavenged by increasing of SOD and CAT activities and results in a reduction of the lipid peroxidation (12). In this investigation, MDA decreased significantly in HO, encapsulated CPECs, and combination therapy groups in comparison with RA+MCAO group, and combination therapy effect was more than HO and encapsulated CPECs alone. 

In one study, it was shown that the pretreatment with HO decreases infarct volume, neurologic deficits scores, and mortality and increases CAT and SOD activities in an animal model of stroke. This shows that HO partly exerts its effects via the increase in antioxidant enzyme activities. It is stated that ROS and HO are compounds that pretreatment via them can increase the activity and expression of SOD (11). Our results showed that HO increased SOD and CAT activities and decreased lipid peroxidation, also. Aliaghaei and colleagues showed that encapsulated CPECs transplants in Alzheimer’s disease animal model improved long-term memory, decreased apoptosis, migration microglia, and gliosis and increased neurogenesis, and the SOD activity (24). The view is that CPECs induce the Nrf2/ARE pathway and antioxidant enzymes overactivation in order to protect neurons against oxidative stress (22). In our study, encapsulated CPECs transplants alone and, in combination with HO, could decrease MCAO-induced cerebral infarct volume by increase SOD and CAT antioxidant enzyme activity. Moreover, encapsulated CPECs decreased the MDA amount. 

Matsumoto et al. showed that CPECs considerably secreted diffusible factors that repressed ischemic brain injury (16). Previous studies show exogenous BDNF, one of CPECs diffusible factors, decreases brain damage volume, and improves behavioral function significantly after acute ischemia (36, 37). Moreover, decreased expression of BDNF is associated with the sensitivity to stress and enhanced stress responses (38). It is also shown that BDNF heterozygous mice are more vulnerable to stress than control mice, revealing behavioral desperation after mild handling stress (39). In the other study, was reported when GDNF introduced to the brain following ischemic stroke, showed neuroprotective effects (15). It seems that encapsulated CPECs presumably indirectly by secreting neurotrophic factors decrease oxidative stress occurred by reperfusion and preconditioning with HO in the ischemic brain. However, the precise mechanism linking combination therapy of HO and CPECs to focal ischemia-reperfusion injury still remains an open question. Several limitations of this study are that measurements were made only up to 24 hours after stroke, and the replication in a second species and sex and age differences were not considered. In a study, Lan et al. found that HO did not reduce infarct size in hypertensive Spraque-Dawley rats (40). Thus, it is suggested that the effect of HO and CPECs also be investigated for a period longer and on the ill animals by considering sex and age. Also, in this work, we did not measure the secreted factors of CPECs but suggest that they should be assayed in future studies.

## Conclusion

The result of this study showed the combination therapy of HO and encapsulated CPECs for ischemic brain damage can be more effective than the HO and encapsulated CPECs alone, and signs decrease of ischemic stroke may relate to an increase in antioxidant enzyme activity. Our study introduces a new method of combination therapy for stroke; We hope that with further researches, the arrival of this combination therapy to the clinic more quickly.
